# Institutional Practices Drive Antibiotic Variability in Neonatal Intensive Care Units: Baseline Evidence to Inform National Stewardship Interventions in Oman

**DOI:** 10.3390/antibiotics15010091

**Published:** 2026-01-16

**Authors:** Abdullah Alqayoudhi, Manoj Malviya, Sathiya Murthi, Mohammed Rasik NV, Adil Said Al-Wahaibi, Raya Al-Habsi, Said Al-Balushi, Talal Alwardi, Agha Hatif Shamsi, Halah Bait Raidan, Aamera Al-Majrafi, Preethi Kiran, Eyad Hani Abu Abu Alhaijaa, Kawther Al Amri, Khalfan Al Abdali, Mohammed S. Al Reesi, Nasser Al-Shafouri, Amal Al-Jabri, Sachin Shah, Said Al-Kindi, Zubair H. Aghai, Mohammed Al-Yahmadi, Amal Al-Maani

**Affiliations:** 1 Centre for Disease Prevention and Control, Ministry of Health (MOH), Muscat 112, Omanadilwahaibi@gmail.com (A.S.A.-W.); amalsaifalmaani@gmail.com (A.A.-M.); 2 Medical City for Military and Security Services, Muscat 124, Omaneyadabualhijaa@gmail.com (E.H.A.A.A.); alamrikas@gmail.com (K.A.A.); sasa_alkindi@yahoo.com (S.A.-K.); 3 Oman Medical Specialty Board, Statistics, Muscat 132, Oman; sathiya.m@omsb.org; 4 Directorate General of Information & Technology (DGIT), Ministry of Health (MOH), Muscat 100, Oman; mohammed.razik@moh.gov.om; 5 Ibra Hospital, Ministry of Health (MOH), Ibra 413, Oman; ryyalhabsi@yahoo.com; 6 Sohar Hospital, Ministry of Health (MOH), Sohar 311, Oman; dr.saeed86@hotmail.com (S.A.-B.);; 7 Nizwa Hospital, Ministry of Health (MOH), Nizwa 611, Oman; talalalwardi83@gmail.com (T.A.);; 8 Sur Hospital, Ministry of Health (MOH), Sur 411, Oman; mishalsuhaa2006@gmail.com; 9 Sultan Qaboos Hospital, Salalah 211, Oman; dr.halaraidan@gmail.com; 10 Rustaq Hospital, Ministry of Health (MOH), Rustaq 318, Oman; aameraalmajarafi@gmail.com; 11 Ibri Hospital, Ministry of Health (MOH), Ibri 511, Oman; alshafouri@gmail.com; 12 Khoula Hospital, Ministry of Health (MOH), Muscat 112, Oman; amal.jabri@gmail.com (A.A.-J.); yahmedy333@hotmail.com (M.A.-Y.); 13 Surya Mother and Child Superspeciality Hospital, Pune 411057, India; sshahdoc@gmail.com; 14 Division of Neonatology, Department of Pediatrics, Thomas Jefferson University, Nemours Children’s Health, Philadelphia, PA 19107, USA; zaghai@nemours.org

**Keywords:** newborn, antibiotics, stewardship, variability, NICU, antimicrobial consumption

## Abstract

**Background**: Antibiotic overuse in Neonatal Intensive Care Units (NICUs) is a major contributor to antimicrobial resistance and adverse neonatal outcomes. This study aims to evaluate baseline antibiotic utilization (AU), identify factors influencing variability, and assess the impact of neonatal characteristics and sepsis incidence. **Methods**: A multicenter retrospective analysis examined AU in seven NICUs from 2019 to 2023, involving 25,532 neonatal admissions during national antibiotic stewardship program implementation. Data encompassed neonatal clinical parameters, sepsis incidence, and AU metrics, including days of therapy (DOT) per 1000 patient-days. Statistical analyses included correlation assessments and multivariate regression to identify determinants of antibiotic use. **Results**: Overall, 43.8% of neonates received antimicrobials, with individual NICUs ranging from 24% to 73% (*p* < 0.001). Antimicrobial-exposed neonates had a mean gestational age of 35.1 weeks [SD ± 4.4] and a mean birth weight of 2360 g [SD ± 970]. Antimicrobial-exposed neonates were generally more premature [35.1 (±4.4) weeks vs. 37.5 (±2.5) weeks (*p* < 0.001)] and had lower mean birth weight [2360 g (±971) vs. 2817 g (±686) (*p* < 0.001)] compared to those not exposed to antimicrobials. Total antimicrobial days varied markedly (8761 to 37,683 days), with DOT per 1000 patient-days ranging from 322 to 1031. Antimicrobial use for culture-negative sepsis varied widely among centers, from 23% to 73%. Antimicrobial-exposed neonates had higher all-cause mortality compared to those who did not [(7.5% vs. 3.2%), (*p* < 0.001)]. Multivariate analysis revealed individual NICU practice patterns remained significant predictors after adjusting for neonatal characteristics. **Conclusions**: Neonatal antimicrobial use varied significantly among NICUs, driven primarily by institutional practices rather than neonatal demographics. These findings provide nationally representative baseline data to inform neonatal antimicrobial stewardship interventions and offer transferable lessons for other countries seeking to optimize antibiotic use in NICUs amid rising global antimicrobial resistance.

## 1. Introduction

Neonatal sepsis is one of the leading causes of morbidity and mortality globally, accounting for nearly three million deaths annually within the first two months of life [1,2]. It is estimated that in low- and middle-income countries (LMIC), about one in four neonatal deaths is due to sepsis, with sepsis-attributable neonatal mortality rates ranging from 10% to 29% [3,4,5]. The disproportionately high mortality in LMIC is primarily caused by diagnostic uncertainty, limited microbiological confirmation, and frequent empiric antibiotic use in the absence or limited use of antibiotic stewardship [6].

Antibiotics are the most commonly prescribed medications in the Neonatal Intensive Care Unit [NICU]; approximately 60–72% of admitted neonates receive antibiotics [7,8,9]. Moreover, up to 30–50% are inappropriate [10,11]. However, overuse contributes to antimicrobial resistance, microbiome disruption, and adverse outcomes, including mortality, necrotizing enterocolitis, prolonged hospital stays, increased health care costs, and abnormal neurodevelopment in extremely low gestational-age neonates [12,13,14,15,16,17]. Data are emerging that early life antimicrobial exposure can lead to potential long-term adverse consequences, such as obesity, allergies, autoimmunity, and other diseases [18,19]. This underscores the critical importance of appropriate antibiotic management in NICUs.

Global antimicrobial resistance trends highlight the urgency of optimizing antibiotic use in all clinical settings, including neonatal care. The WHO GLASS 2025 report shows that resistance to common antibiotics increased significantly: from 2018 to 2023, over 40% of monitored pathogen–antibiotic pairs exhibited rising resistance, particularly in the Eastern Mediterranean and South-East Asia, where about one in three infections was resistant to first-line drugs [20]. This data emphasizes that antimicrobial resistance is widespread and uneven, with higher rates in regions with weak surveillance systems. The GLASS report further highlights that many countries struggle to generate high-quality data on AMR and antibiotic use, which complicates benchmarking and improving antibiotic prescribing practices. Global patterns of antibiotic use reveal regional and income-related variability, with few countries meeting targets for the proportionate use of the ‘Access’ group of antibiotics to reduce resistance. These global trends—rising resistance across diverse pathogens and uneven stewardship infrastructure—have clear implications for neonatal care. Neonates are uniquely vulnerable to infection and frequently exposed to broad-spectrum antibiotics, yet data on resistance patterns and appropriate empiric therapy remain limited in many settings. This evolving global AMR landscape heightens the need for robust surveillance of antibiotic use and resistance in NICUs and informs the rationale for studying institutional drivers of antibiotic utilization.

There is a substantial unexplained variability in antibiotic prescribing across NICUs. A landmark study reported remarkable heterogeneity, noting a 40-fold variation in antibiotic use across US NICUs despite similar culture-proven sepsis burdens [21]. While neonatal factors such as gestational age, birth weight, and sepsis incidence logically influence antibiotic utilization, multiple studies demonstrate these clinical variables explain only a portion of observed practice variation [21,22]. Similarly, studies demonstrated wide heterogeneity in antibiotic prescribing practices independent of clinical case mix [22,23]. These findings suggest that institutional factors, such as prescribing culture, availability of antimicrobial stewardship programs [ASPs], and variability in clinical decision-making, may have a stronger influence on antibiotic utilization than clinical characteristics of neonates. In the Middle East, data evaluating neonatal antibiotic utilization are limited, and there is a lack of national benchmarking to inform stewardship interventions [24,25]. Understanding local utilization patterns is essential, particularly in the context of rising AMR rates and evolving stewardship initiatives across the region.

Recent studies have shown that neonatal antimicrobial stewardship programs can safely reduce antibiotic exposure without compromising clinical outcomes, highlighting the importance of understanding drivers of antibiotic use at the institutional level [26,27,28,29].

Oman has introduced a national antimicrobial stewardship program; however, the extent to which clinical characteristics and institutional practices influence antibiotic variability in neonatal care has not previously been evaluated.

Therefore, the objectives of this study were to:

1. Quantify baseline antibiotic consumption across seven NICUs in Oman;
2. Assess the influence of neonatal demographics and culture-positive sepsis incidence on antibiotic utilization;
3. Determine whether variability in antibiotic use is primarily driven by neonatal characteristics or by institutional practice patterns. By identifying key determinants of antibiotic variability, this study provides critical evidence to inform national stewardship policy and optimize antibiotic prescribing practices in neonatal care.

This study will provide the first national, multicenter epidemiologic assessment of neonatal antimicrobial use in Oman and one of the few such evaluations from the Middle East. By linking antibiotic use metrics to neonatal characteristics and institutional factors during the initial implementation of a national antimicrobial stewardship program, the study will establish baseline benchmarking data essential for assessing stewardship impact over time. The findings are relevant beyond Oman, as they address a universal challenge faced by NICUs worldwide: significant variability in antibiotic use driven by institutional practices rather than disease burden, especially in settings that lack standardized neonatal stewardship frameworks.

## 2. Results

### 2.1. Baseline Neonatal Characteristics

A total of 25,532 neonatal admissions from seven NICUs were evaluated. The baseline characteristics of the entire cohort shown in Table 1. Antimicrobial-exposed neonates had a mean gestational age of 35.1 weeks [SD ± 4.4] and a mean birth weight of 2360 g [SD ± 970]. Antimicrobial-exposed neonates were generally more premature [35.5 (±4.4) weeks vs. 37.5 (±2.5) weeks (*p* < 0.001)], had lower mean birth weight [2360 g (±971) vs. 2817 g (±686) (*p* < 0.001)], and were more likely to be born by cesarean section compared to those not exposed to antimicrobials, [(51% vs. 46%), *p* < 0.001]. More males received antimicrobials than the non-antimicrobials group [(57% vs. 54%), (*p* < 0.001)]. Both 1 min (AS-1) and 5 min (AS-5) APGAR scores were significantly lower in the antimicrobials cohort (*p* = 0.000 for both). The median AS-1 was 8 (IQR 6,8) for the treated group versus 8 (IQR 7,9) for the untreated group, and the median AS-5 was 9 (IQR 8,9) versus 9 (IQR 9,10), respectively. Antimicrobial-exposed neonates had an increased all-cause mortality compared to those who did not [(7.5% vs. 3.2%), (*p* < 0.001) presented in Table 2. Mortality data for individual NICUs for neonates who received antimicrobials versus those who received no antimicrobials are presented in Table 3.

### 2.2. Baseline Characteristics of Individual NICU Patients Who Received Antimicrobials in the Enrolled NICUs

The baseline characteristics of the 11,171 patients who received antimicrobials varied significantly across the seven NICUs (Table 2). NICU A and NICU D had low mean GA (34.5 and 34.6 weeks, respectively), suggesting relatively premature populations. NICU D, which had the highest antimicrobial utilization rate (73%), also reported the lowest mean BW (2286 gm) among the centers. The Cesarean section rate varied significantly among the antimicrobial recipients, ranging from a low of 23% in NICU A to a high of 55% in NICU C [*p* < 0.001].

**Table 2:** Baseline characteristics of individual NICU patients who received antimicrobials in the enrolled NICUs.

NICUS	A	B	C	D	E	F	G	Total	*p*-Value
Total NICU admissions	5584	4330	2192	4090	2937	3551	2848	25,532	
Number of patients who received antimicrobials n, (%)	1327 (24)	1990 (46)	1554 (71)	2999 (73)	1317 (45)	1256 (35)	728 (25)	11,171 (43.8)	0.001
BW in gm. mean (SD)	2263 (1031)	2483 (930)	2413 (901)	2286 (966)	2451.29 (980)	2317 (1000)	2309 (998)	2360 (970)	0.01
GA Mean (SD)	34.5 (5)	35 (4)	35.6 (4)	34.6 (4)	35.7 (4)	34.7 (5)	34.9 (5)	35.1 (4)	0.01
Male n (%)	717 (54)	1215 (61)	883 (57)	1747 (58)	754 (57)	702 (56)	409 (56)	6427 (58)	<0.001
AS-1 Median (IQR)	8 (6,8)	7 (7,8)	6.6 (5,8)	8 (6,8)	8 (9,9)	7 (7,9)	6.5 (6,8)	8 (6,8)	<0.001
AS-5 median (IQR)	9 (9,10)	9 (9,9)	8 (8,9)	8 (8,9)	8 (8,10)	9 (9,10)	8.2 (8,9)	9 (8,9)	<0.001
C-section n/Total (%)	614/2646 (23)	956/2169 (48)	863/1554 (55)	1786/2999 (49)	569/1317 (43)	545/1256 (43)	348/728 (48)	5490 (49)	<0.001

BW = birth weight, GA = Gestational Age, gm = grams, AS-1 = APGAR Score at 1 min, AS-5 = APGAR Score at 5 min, IQR = Interquartile range.

### 2.3. Antimicrobial Usage and Variability

Overall, 43.8% of neonates received at least one antimicrobial; NICU-specific rates ranged from 24% to 73%, as shown in Table 3, with individual NICUs’ number of patients who received antimicrobial as: NICU A [24%], NICU B [46%], NICU C [71%], NICU D [73%], NICU E [45%], NICU F [35%], and NICU G [25%]. Total antibiotic days ranged from 12,793 in NICU E to 37,683 in NICU D. The total antimicrobial utilization rate (DOT) across all NICUs was 654. This rate varied widely, ranging from the lowest consumption rate in NICU A (322 DOT) and NICU F (361 DOT), to the highest rates in NICU C (1031 DOT) and NICU D (897 DOT) (*p* < 0.001).

Ampicillin and gentamicin were the most used antibiotics across NICUs. Ampicillin was administered to 90% of patients on antimicrobials, with usage rates ranging from 81% in NICU A to 94% in NICU C (*p* < 0.001). The percentage of patients on Ampicillin for more than three days varied from 34% in NICUs A and F to 50% in NICU G. Ampicillin consumption measured by DOT also showed significant variability, ranging from 101 (NICU A) to 362 (NICU C) (*p* < 0.001).

Gentamicin was the main aminoglycoside, given to 84% of antimicrobial-treated patients. Its use varied from 43% (NICU C) to 92% (NICUs A, B, E) (*p* < 0.001). Its consumption ranged from 83 DOT (NICU F) to 251 DOT (NICU B) (*p* < 0.001).

Amikacin use showed the greatest variation in usage patterns across units, with only 1% of NICU B patients receiving it, compared with 60% in NICU C (*p* < 0.001). Consumption ranged from 4 DOT in NICU B to 170 DOT in NICU C (*p* < 0.001), with NICU C favoring Amikacin over Gentamicin.

BSA with [meropenem, piperacillin tazobactam, vancomycin, cefotaxime] was used in 25% of treated patients, ranging from 10% (NICU E) to 32% (NICU D) (*p* < 0.01). BSA consumption, measured by DOT, showed significant variability, ranging from 71 (NICU E) to 315 (NICU C) (*p* < 0.001).

Neonatal sepsis (blood culture positive) was identified in 4.4% (1134/25,532) of the overall cohort, with notable differences across NICUs, ranging from 3.4% (NICU A) to 10.2% (NICU C) (*p* < 0.01). Gram-positive infections accounted for 67.5% (791/1134), Gram-negative for 29.5% (324/1134), and *Candida* species for 1.6% (19/1134). Among Gram-positive pathogens, CONS accounted for 60% (474/791), *Streptococcus agalactiae* 12% (97/791) *S. aureus* 6.5% (50/791) and other Gram-positive species 21.5%. The main Gram-negative bacteria included *Klebsiella* spp. 26.8% (87/324), *Pseudomonas* spp. 22.8% (74/324), *E. coli* 16.6% (50/324), *Serratia* spp. 14.8% (48/324), *Acinetobacter* spp. 4.6% (15/324), and other organisms at 14.2% (46/324).

A high percentage of infants with blood culture-negative [BCN] status received antibiotics, ranging from 23% to 73%. (NICU A = 23%, NICU B = 46%, NICU C = 71%, NICU D = 73%, NICU E = 45%, NICU F = 35%, NICU G = 26%).

**Figure 1:**
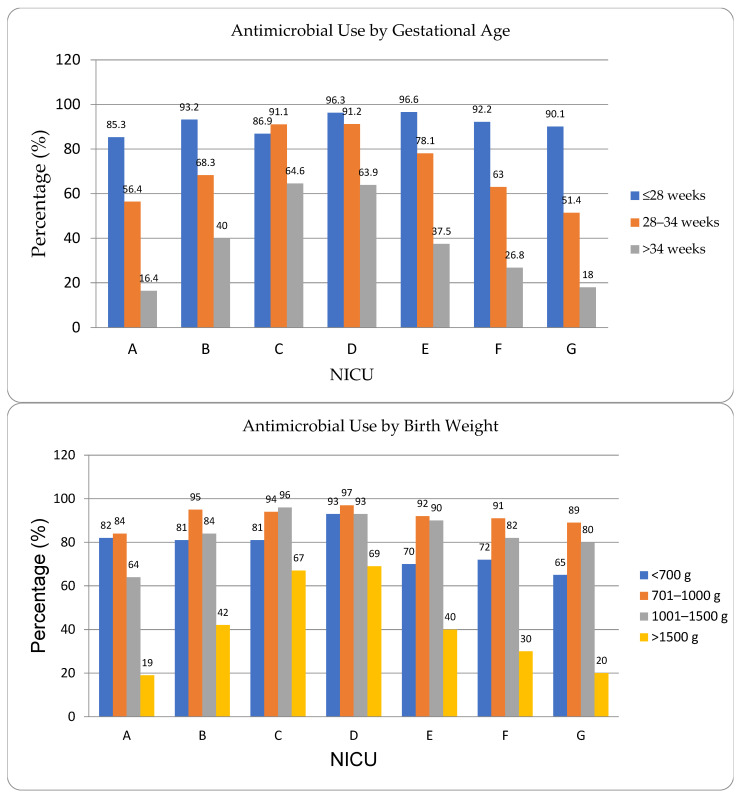
Antimicrobial Use by Gestational and Birth Weight Across NICUs.

Antimicrobial use per birth weight and gestational age is shown in Figure 1.

Analysis of antimicrobial use [AU] across birth weight groups showed higher use in lower birth weight groups: <1000 g = 93%, 1000–1500 g = 84%, >1500 g = 39%, Figure 1. Infants [>1500 g] exhibited the highest inter-institutional variability [19% to 69%].

**Table 3:** Antimicrobials use over the study years in seven neonatal intensive care units.

NICU Names	A	B	C	D	E	F	G	Total	*p* ^a^ Value
Total Admissions ^b^	5584	4330	2192	4090	2937	3551	2848	25,532	
Total Patients AU n (%)	1327 (24)	1990 (46)	1554 (71)	2999 (73)	1317 (45)	1256 (35)	728 (25)	11,171 (44)	<0.001
Total antimicrobial days	13,098	23,196	17,680	37,683	12,793	10,022	8761	123,233	<0.001
DOT ^c^	322	886	1031	897	765	361	492	654	<0.001
Ampicillin ^d^ n (%)	1077 (81)	1781 (89)	1456 (94)	2701 (90)	1223 (93)	1155 (92)	659 (90)	10,052 (90)	<0.001
Ampicillin ^d^ days	4103	7821	6213	2373	5432	3975	2641	20,721	<0.001
Ampicillin ^d^ DOT ^c^	101	299	362	295	325	143	148	110	<0.001
Ampicillin > 3 days n (%)	369 (34)	638 (36)	658 (45)	-	-	388 (34)	332 (50)		-
Gentamicin n (%)	1225 (92)	1827 (92)	666 (43)	2721 (91)	1217(92)	1075 (86)	661 (91)	9392 (84)	
Gentamicin Days	4295	6569	2339	10,055	4132	2293	2116	31,799	<0.001
Gentamicin DOT	106	251	136	239	247	83	119	169	<0.001
Amikacin n (%)	160 (12)	22 (1)	925 (60)	448 (15)	27 (2)	139 (11)	31 (4)	1752 (16)	
Amikacin Days	507	105	2922	1961	100	419	166	6180	<0.001
Amikacin DOT	12	4	170	47	6	15	9	33	<0.001
BSA, n (%)	288 (22)	521 (26)	442 (28)	956 (32)	128 (10)	288 (23)	198 (27)	2821 (25)	<0.01
BSA days	3218	6373	5406	11,114	1182	2700	2512	32,505	<0.001
BSA DOT	79	243	315	265	71	97	141	173	<0.001
Others n, (%)	201 (15)	567 (28)	178 (11)	904 (30)	132 (10)	150 (12)	231 (32)	2360 (21)	<0.003
Other days	1204	4716	1177	6481	784	890	2110	17,362	<0.001
Other DOT	30	180	69	154	47	32	118	92	<0.001
Sepsis ^e^	190 (3.4)	225 (5.2)	224 (10.2)	275 (6.7)	-	131 (3.7)	102 (3.6)	1134 (4.4)	<0.01
Surgery n (%)	261 (4.6)	57 (1.3)	27 (1.1)	103 (2.5)	126 (4.3)	70 (2.0)	17 (1)		-
Mortality ^f^ (%)	7.2 vs. 1.8	5.9 vs. 2.5	8.3 vs. 6.6	8.6 vs. 4.5	6.3 vs. 2	7.4 vs. 1.8	8.5 vs. 1.4	7.5 vs. 2.3	0.000

^a^ = *p*-value denotes comparison among NICUs, ^b^ = admission to NICU, DOT = day of therapy, ^c^ = per 1000 patient-days, ^d^ = ampicillin plus penicillin, ^e^ = blood culture positive, BSA = broad-spectrum antibiotics (meropenem, Piperacillin tazobactam, vancomycin, cefotaxime), Others = Amphotericin, flucanazole, ceftazadine, metronidazole, cloxacillin), ^f^ = neonates with antimicrobial use vs. not use.

### 2.4. Influence of Neonatal and Institutional Factors

Higher sepsis rates correlated with increased antibiotic use [r = 0.45, *p* < 0.01]. Neonates with lower GA and birth weight received more antibiotics, but these factors did not fully account for inter-NICU differences in utilization rates. Multivariate regression revealed that NICU site remained a significant independent predictor of antibiotic days [*p* < 0.001] after comprehensive adjustment for gestational age, birth weight, Apgar scores, cesarean section rates, surgery, and documented sepsis incidence, indicating that practice patterns and institutional culture strongly influence prescribing behaviors.

## 3. Discussion

This multicenter study provides the first nationally representative baseline assessment of antibiotic utilization across seven NICUs in Oman, revealing substantial inter-institutional variability driven primarily by institutional practices rather than patient characteristics. Our findings demonstrate a 3-fold variation in antibiotic utilization rates [24% to 73%] and a 3.2-fold difference in antibiotic intensity [322 to 1031 DOT per 1000 patient-days] across institutions. Most significantly, multivariate analysis confirmed that NICU-specific practice patterns remained strong independent predictors of antibiotic use even after controlling for neonatal characteristics, sepsis burden, and other clinical factors.

These findings have significant implications for antimicrobial stewardship in Oman and beyond. The variability across units indicates opportunities for optimization through unit-specific interventions. The continued influence of practice patterns after case-mix adjustment highlights the need for targeted strategies that address local prescribing cultures and protocols.

### 3.1. Variability in Context: Global and Regional

Our observed variability aligns with findings from major international studies. The NeoOBS study, conducted across 19 sites in 11 countries (mainly Asia and Africa), documented 206 empiric antibiotic regimens. Only 25.9% of infants received WHO “Access” group of antibiotics; many centers used medium- or high-watch regimens such as piperacillin-tazobactam and carbapenem [4]. Schulman et al. reported up to 40-fold variation in antibiotic use across 127 USA NICUs [2.4% to 97.1% of patient-days], despite similar culture-proven sepsis burdens [21]. Similarly, a point-prevalence survey of 240 NICUs across 41 countries found that 89.2% of neonates received antibiotics, with substantial inter-institutional variation: ampicillin plus gentamicin was used in only 44% of centers, and 31 different antibiotic combinations were employed for early-onset sepsis [22]. Another study involving 51 NICUs across the USA showed that patient complexity accounted for only part of the variation in AU, with local guidelines, stewardship activities, and institutional culture playing crucial roles [23]. Our 3-fold variation, while significant, falls within the range reported globally, suggesting that practice heterogeneity is a universal challenge in neonatal care.

Limited data exists on antibiotic consumption patterns in Middle Eastern NICUs, making our study particularly valuable for regional benchmarking. Regional surveys consistently document higher broad-spectrum antibiotic use and elevated resistance rates compared to developed countries, often reflecting local epidemiology and empiric prescribing practices adapted to regional pathogen patterns [24,25].

Our study found a significant variation [DOT of 71 to 315] in broad-spectrum antibiotic use [meropenem, piperacillin-tazobactam, vancomycin, cefotaxime]. Our observed institutional variability likely reflects different approaches to broad-spectrum empiric therapy, similar to those reported in European surveys [22], particularly for late-onset sepsis and in high-risk populations. The NeoOBS study found that 41.8% of neonates received Watch or Reserve group antibiotics as empiric therapy, despite WHO recommendations favoring Access group agent [4]. Regional data from Saudi Arabia showed similarly high rates of broad-spectrum antibiotic consumption, with mean DOT exceeding 400 per 1000 patient-days in some pediatric and neonatal ICUs [24]. NICUs with higher overall utilization may demonstrate more liberal use of broad-spectrum agents, longer durations of empiric broad-spectrum coverage, or different de-escalation practices following culture results.

### 3.2. Culture-Negative Sepsis and Antimicrobial Utilization

In this study, a significant proportion [23–71%] of infants with blood culture-negative [BCN] status received antibiotics. Culture-negative sepsis management represents a critical driver of antibiotic overuse globally and a high-yield stewardship target. Studies have found that most infants receiving prolonged antibiotic courses have negative cultures, yet continuation rates vary significantly across institutions [30,31]. A large international study across 11 countries (Europe, North America, and Australia) found that 37% of late-preterm and term newborns receiving IV antibiotics in the first week had culture-negative sepsis treated for ≥5 days, significantly increasing total antibiotic days [31]. Among very preterm infants, a multicenter study found 89.4% of antibiotic courses for suspected late-onset sepsis were culture-negative, yet these cases received a median of 7 days of therapy [32].

Our data suggest culture-negative sepsis practices likely contribute significantly to observed differences, with higher-utilization NICUs potentially having more liberal continuation policies or different evaluation thresholds. Recent quality improvement initiatives demonstrate that targeting culture-negative sepsis can substantially reduce exposure by implementing early discontinuation protocols [33,34]. A single-center study implementing a defined antibiotic stop policy for culture-negative healthcare-associated infections reduced antibiotic overuse from 14.49 to 3.26 days per 1000 patient-days with no increase in mortality, need for antibiotic restart, or adverse outcomes [35]. In another quality-improvement initiative, implementing prioritized case review and weekly stewardship resulted in an 81% reduction in antibiotics DOT for culture-negative sepsis with no adverse balancing outcomes [34].

The most common justifications presented for continuing antibiotics despite a negative blood culture include abnormal laboratory values, clinical instability, and provider concern—factors with limited specificity for bacterial infection [11]. This diagnostic ambiguity presents a significant opportunity for stewardship interventions that focus on explicit discontinuation criteria.

### 3.3. Risk Stratification and Stewardship Implications

Our findings confirm the expected inverse relationship between birth weight/gestational age and antibiotic utilization [AU < 1000 g; 93%], consistent with international observations that prematurity and low birth weight are primary drivers of sepsis evaluations and antibiotic exposure [10]. A systematic review of antimicrobial stewardship in premature infants found that antibiotic use was highest among extremely preterm infants (<28 weeks) and that prolonged empiric therapy was common in infants ≤34 weeks gestation [36]. A recent study involving 36,701 infants weighing less than 1000 g across 402 US NICUs found that antibiotic exposure remained relatively high but stable, at 89.9% in 2009 and 89.3% in 2021, over 13 years [37]. This gradient reflects both higher infection risk in more premature infants and lower clinician tolerance for withholding antibiotics in the face of diagnostic uncertainty. However, a recent study from China showed that restricting antibiotic use in VLBW infants is associated with improved short-term clinical outcomes and can be achieved safely and effectively [38].

### 3.4. Impact of the Presence of Structured ASP and Neonatologist at the Frontline in NICU

The differential presence of structured antimicrobial stewardship programs (ASP) and neonatologist staffing across the seven Omani NICUs in our study represents important contextual factors that may influence antimicrobial prescribing patterns. It also provides a natural experiment to examine the impact of these organizational factors on antibiotic utilization.

Recent studies provide strong evidence that structured, multifaceted ASPs can significantly reduce antibiotic use without compromising safety and may even improve outcomes. A meta-analysis of 70 studies involving more than 350,000 neonates found that structured stewardship interventions effectively reduce antibiotic use without increasing adverse events [27]. Individual AS program reports document even larger reductions: a Lebanese tertiary NICU achieved a 30% reduction in antibiotic use, sustained over five years [39], while a Canadian program reduced DOT from 472 to 313 per 1000 patient-days over a decade [40].

Most traditional ASPs are led by an infection control or microbiologist and are resource intensive. A neonatologist-driven ASP is relevant in settings where dedicated AS experts (infectious disease experts, microbiologists, or pharmacists) or stewardship resources are limited. Several successful ASPs that are primarily led by neonatologists have been reported [11,41]. Oman’s experience reported by Malviya et al. (2024) described a neonatologist-driven ASP that achieved reductions in antibiotic use through the development of unit-specific guidelines, regular case reviews, and educational initiatives—all led by neonatology staff rather than external stewardship teams [11]. Neonatologists’ expertise in neonatal infection epidemiology, pharmacokinetics, and outcomes can lead to more appropriate prescribing practices. However, this must be balanced against the risk of specialty-specific prescribing cultures that may sustain high medication use. The lack of dedicated neonatologists in one unit in our study may have contributed to prescribing variability. However, whether this results in higher or lower use cannot be determined without analyzing that unit’s specific prescribing patterns. Regardless of whether neonatologists are present, successful stewardship requires multidisciplinary engagement, including pharmacy, microbiology, infectious diseases specialist, nursing, and unit leadership. Robust evidence has established that multidisciplinary team-led antimicrobial stewardship programs can safely reduce antibiotic use without increasing adverse outcomes, including sepsis-related mortality, necrotizing enterocolitis, or length of stay, particularly when stewardship strategies are embedded into routine clinical decision-making and include regular audit and feedback mechanisms [29,42].

### 3.5. Implications for Clinical Practice: Local, Regional, and Global

This study provides the first nationally representative baseline assessment of antibiotic utilization across Oman’s seven NICUs, generating data with important implications at local, regional, and global levels. By establishing baseline antibiotic use metrics and identifying institutional drivers of variability, this study provides a foundation for evaluating the impact of stewardship interventions over time and contributes to global efforts to optimize antibiotic use in neonatal care. The findings contribute to the limited but growing literature on neonatal antibiotic use in the Middle East and Gulf region, while offering transferable lessons for other countries seeking to establish antimicrobial stewardship programs amid rising global resistance. It allows regional benchmarking, identifying institutional drivers of variability, and recognizes common challenges in Middle Eastern healthcare contexts.

Our findings underscore the need for strengthened antibiotic stewardship in neonatal settings, aligning with the World Health Organization’s Global Antimicrobial Resistance and Use Surveillance System [GLASS] 2023 report, which highlights that antimicrobial resistance [AMR] was most frequent in the South-East Asia and Eastern Mediterranean regions, reaching 31.1%—almost one in three infections [WHO, GLASS 2023] [20].

Our study guides national stewardship priorities. The specific patterns observed—particularly the high proportion of antibiotic use for culture-negative sepsis, ampicillin utilization patterns, broad-spectrum antibiotic use, and the persistence of practice pattern effects after case-mix adjustment—point to concrete priorities for Oman’s national stewardship efforts. Priority interventions should include:

(1) Establishment of multidisciplinary stewardship teams in units currently lacking structured programs;
(2) Development and dissemination of national guidelines for empiric antibiotic therapy in neonates, with explicit initiation criteria and maximum durations for culture-negative cases;
(3) Implementation of standardized DOT reporting across all NICUs to enable ongoing monitoring and benchmarking;
(4) Creation of a national learning collaborative to facilitate knowledge sharing between units and rapid dissemination of effective practices;
(5) Leveraging existing infrastructure: having one NICU with a well-established structured ASP offers a proven internal model and resources that can support scaling up nationally. Each unit should adapt interventions to the local context, prescribing patterns, and available resources.

Relevance to low and middle-income countries (LMIC): Several aspects of our findings are particularly relevant to other LMIC. Substantial antibiotic overuse exists and is modifiable even in settings with limited stewardship infrastructure. Second, low-cost, clinician-led stewardship models can be effective without requiring extensive additional resources, and finally, focusing on culture-negative sepsis duration offers high-yield opportunities for rapid reductions in unnecessary exposure

### 3.6. Strengths, Weaknesses, and Novelty

Major strengths include a multicenter, nationally representative design that captures practice variation across seven NICUs, with a large sample size of 25,532 neonatal admissions. The extended timeframe of study captures variations across years, providing more stable estimates than point-prevalence surveys or shorter observational periods. The use of standardized DOT metrics that enable international benchmarking, comprehensive data on neonatal characteristics and institutional factors that support multivariable analysis, and the timing of national stewardship implementation provide essential baseline data for future evaluation.

Key limitations include the retrospective design, which may introduce misclassification bias, and the limited availability of outcome data beyond mortality. We did not evaluate the influence of local staffing patterns, diagnostic stewardship levels, sepsis biomarker availability, the extent of antibiotics stewardship implementation, or antibiotics consumption monitoring on antimicrobial use. There is a risk of residual confounding, as unmeasured factors such as illness severity, central venous lines, mechanical ventilation, and comorbidities might partially explain the differences in antibiotic use observed. Using validated severity scores would enhance causal inference.

In summary, this study combines the methodological strengths of a multicenter design, a large sample size, and standardized metrics with novel contributions, including first national Omani data, regional evidence from an understudied area, and an explicit focus on modifiable practice variation.

## 4. Materials and Methods

### 4.1. Study Design and Setting

This was a retrospective, multicenter observational study conducted across seven level III neonatal intensive care units [NICUs] in Oman from January 2019 to December 2023, during the implementation of the national antimicrobial stewardship program. The participating NICUs were selected from secondary care regional hospitals across different districts with similar capacities and scopes of care. These NICUs admit high-risk neonates [Level II–III] and provide complete intensive care services except for surgical interventions. The annual admissions range between 700 and 1200 infants per unit. We randomly assigned labels from A to G to the NICUs. All NICUs have dedicated certified neonatologists except Unit C. One unit (NICU A) had a dedicated neonatal antibiotic stewardship program [ASP], whereas the remaining NICUs operated without NICU-specific protocols during study period. A team of neonatologists appointed by the Directorate of Infection Surveillance and Control (DISC) Ministry of Health, Muscat, Oman, who had training and experience in successfully implementing ASP (Malviya et al.), conducted in-person visits and stayed for one week at each participating NICU. In addition, DISC team leaders visited and spent the entire day meeting with all stakeholders. During each visit, the team conducted comprehensive evaluations of existing ASP activities.

### 4.2. Study Population

All neonates admitted to the participating NICUs during the study period were eligible for inclusion. No exclusion criteria were applied based on gestational age or birth weight in order to capture routine antibiotic prescribing practices. A total of 25,532 admissions were evaluated. Neonates admitted for less than 24 h or transferred before complete data collection were excluded from the analysis.

### 4.3. Data Collection and Collection Process

Data were extracted retrospectively from hospital electronic medical records and pharmacy dispensing systems using a standardized data collection template developed centrally and implemented across all sites. Collected variables included neonatal demographic (gestational age, birth weight, Apgar scores), documented sepsis episodes, mortality, antibiotic prescriptions measured as days of therapy (DOT) per 1000 patient-days, and microbiological culture results where available.

In accordance with routine hospital practice, all antibiotic prescriptions were computerized, and antimicrobial consumption for each admitted neonate is available within the health electronic record system and can be tracked using patient identification number or unit code by hospital pharmacies across all participating NICUs. Antimicrobial utilization data for ten antibiotics and two antifungal agents were extracted from the hospital pharmacy database.

To ensure consistency and data quality, data extraction procedures were standardized across sites and supported by written guidance. Extracted datasets were reviewed for completeness and internal consistency. Data validity and reliability were verified through a multi-step process involving two national Information Technology (IT) personnel in collaboration with local IT teams. In addition, two neonatologists independently and randomly cross-checked extracted datasets. Any discrepancies were resolved through consultation with site investigators prior to analysis to ensure accuracy and consistency.

### 4.4. Outcome Measured

The primary outcomes were:

1. The total number of neonates admitted to each NICU who received at least one antibiotic.
2. The antibiotic utilization rate, expressed as DOT per 1000 patient-days.

DOT was defined as the number of days a neonate received a given antimicrobial agent, irrespective of dose or frequency. For example, administration of ampicillin for four days was counted as four DOT. In cases of combination therapy, each antibiotic was counted as a separate DOT for each calendar day. DOT was aggregated at the unit level and standardized per 1000 patient-days to allow comparison across NICUs with differing patient volumes and lengths of stay.

The secondary outcomes included:

1. The incidence of blood culture-positive sepsis,
2. Variation in antimicrobial use among neonates with culture-negative sepsis,
3. Microbial profile of culture-positive sepsis,
4. Mortality in neonates who received antimicrobial agents compared to those who did not.

### 4.5. Statistical Analysis

Descriptive statistics were used to summarize neonatal characteristics, and microbiological findings and antibiotic use patterns. Antibiotic utilization rates were compared across NICUs using rate ratios and graphical visualization. Baseline characteristics were compared across NICUs using ANOVA or the Kruskal–Wallis test for continuous variables and the Chi-square test for categorical variables. Correlation assessments evaluated relationships between institutional factors and antibiotic use. The correlation between neonatal factors, sepsis rates, and antibiotic use was assessed using Pearson or Spearman coefficients. Multivariate linear regression identified independent predictors of antibiotic utilization, controlling for neonatal demographics, sepsis incidence, and surgical interventions. All tests were two-tailed, and a *p*-value < 0.05 was considered significant. All statistical analyses were performed using SPSS v.30.0 [Armonk, NY, USA: IBM Corp]. Ministry of health Ethics Committee approved this investigation (Ethics file# 27200, dated 5 September 2023).

## 5. Conclusions

This multicenter study highlights key patterns in antibiotic use across Omani NICUs and underscores the need for strengthened antimicrobial stewardship in this vulnerable population. Substantial inter-institutional variability in antibiotic utilization, not fully explained by differences in culture-confirmed sepsis burden, suggests that institutional prescribing practices play an important role in shaping antibiotic exposure. The lower antibiotic use observed in a NICU with a dedicated neonatal antimicrobial stewardship program further supports the potential impact of structured, neonatal-specific stewardship interventions. Establishing unified national protocols, enhancing clinician education, and routinely benchmarking antibiotic use can optimize prescribing practices, reduce unnecessary antibiotic exposure, and help mitigate the growing threat of antimicrobial resistance. Together, these findings provide nationally representative baseline data to inform stewardship strategies and are relevant to broader international efforts to improve antibiotic use in neonatal care.

## 6. Future Directions

Future research should examine the clinical results linked to various antibiotic prescribing practices, including trends in antimicrobial resistance. These outcomes may include necrotizing enterocolitis, chronic lung disease, and long-term neurodevelopmental effects. Additionally, studies should explore how early antimicrobial exposure influences the microbiota and impacts health for both short and long-term outcomes. Future research should focus on integrating technology and artificial intelligence, investigate using electronic health records to create automated stewardship alerts and reminders by developing a dynamic antimicrobial consumption dashboard, and employ artificial intelligence-assisted decision support to optimize antibiotic use.

## Figures and Tables

**Table 1:** Baseline characteristics of all neonates admitted to NICUs.

Total NICU Admission: 25,532			
	Antimicrobials Received	No antimicrobials	*p* value
	11,171 (43.8%)	14,361 (56.2%)	
Male n (%)	6423 (57)	7764 (54)	<0.001
BW in gm, mean (SD)	2360 (970)	2817 (686)	<0.001
GA Mean (SD)	35.1 (4.4)	37.5 (2.5)	0.000
Cesarean n (%)	5681 (50.9)	6600 (46)	0.000
AS-1 Median IQR	8 (6,8)	8 (7,9)	0.000
AS-5 Median IQR	9 (8,9)	9 (9,10)	0.000
Hospital Stay Mean (SD)	13.12 (19.8)	2.7 (4.8)	0.000
Mortality (%)	7.5	3.2	0.000

BW = birth weight, GA = Gestational Age, AS-1 = APGAR Score at 1 min, AS-5 = APGAR Score at 5 min. IQR = Interquartile Range.

## Data Availability

The original contributions presented in this study are included in the article. Further inquiries can be directed to the corresponding author.
